# Anti-Inflammatory Effects of Curcumin on Insulin Resistance Index, Levels of Interleukin-6, C-Reactive Protein, and Liver Histology in Polycystic Ovary Syndrome-Induced Rats 

**DOI:** 10.22074/cellj.2017.4415

**Published:** 2017-08-19

**Authors:** Shima Mohammadi, Latifeh Karimzadeh Bardei, Vida Hojati, Azita Ghorbani, Mohammad Nabiuni

**Affiliations:** 1 Department of Animal Biology, Faculty of Biological Sciences, Kharazmi University, Tehran, Iran; 2 Laboratorys Animal Center and Cellular and Molecular Research Laboratory, Faculty of Biological Sciences, Kharazmi University, Tehran, Iran; 3 Department of Biology, Damghan Branch, Islamic Azad University, Damghan, Iran; 4 Department of Cell and Molecular Biology, Faculty of Biological Sciences, Kharazmi University, Tehran, Iran

**Keywords:** PCOS, Liver, Curcumin, Insulin Resistance

## Abstract

**Objective:**

Curcumin protects the liver against injury and fibrosis through suppressing hepatic inflammation, attenuating hepatic oxidative stress (OS), and inhibiting hepatic stellate cells (HSCs) activation. Non-alcoholic fatty liver disease (NAFLD) and polycystic ovary syndrome (PCOS) are considered as common metabolic disorders. Low-grade chronic inflammation with different markers, such as elevated C-reactive protein (CRP) and interleukin-6 (IL-6) levels, play a crucial role in PCOS. This study aimed to evaluate the therapeutic effects of curcumin on IL-6 and CRP levels as well as insulin resistance (IR) index on liver function in PCOS rats.

**Materials and Methods:**

In this experimental study, 90 adult Wistar rats were divided into control (n=18), sham (n=18), PCOS (n=18) and curcumin-treated PCOS groups (n=36). PCOS group was injected subcutaneously with 2 mg estradio-valerate (E2V). After 60 days, PCOS group was treated with curcumin [100 and 300 mg/kg body weight (BW)] for 14 days and anesthetized by chloroform. Blood and liver samples were collected for histological and serological analyses. Data were analyzed using In-Stat 3 via one-way analysis of variance (ANOVA).

**Results:**

Histological and serological analyses showed a reduction in number of necrotic cells, IR index, as well as IL-6 and CRP levels in PCOS rats that were treated with various concentrations of curcumin.

**Conclusion:**

In this study, curcumin decreased liver inflammation by induction of insulin sensitivity and reduction of hepatic necrosis. Therefore, curcumin may be considered as protective factor against inflammatory state of PCOS.

## Introduction

The plants have been used to treat disease for many years, although healing properties and chemical constituents in many of them are still unknown. Various medicinal plants have been used to treat different diseases worldwide (1). Inflammation of liver is among series diseases that has been the focus of plant therapy in recent years. Some plants with therapeutic values are as follows: *Silybum marianum* (milk thistle), *Picrorhiza kurroa* (kutki), *Curcuma longa* (turmeric), *Camellia sinensis* (green tea), and *Glycyrrhiza glabra* (licorice) (2). Curcumin (diferuloylmethane) as a yellow substance in turmeric is obtained from the rhizome of *Curcuma Longa Linn* (Zingiberaceae), a perennial herb distributed mainly throughout tropical and subtropical regions of the world (3). Curcumin modulates the biological activity of many signaling molecules. It has anti-cancer activities attributing to its antioxidant and anti- inflammatory properties (4). Inflammation of liver, oxidative stress (OS), hepatic stellate cells (HSCs) activation and mitochondrial dysfunction are induced following hepatic injury, but it has been shown that curcumin may be protective against these abnormalities (5). Polycystic ovary syndrome (PCOS) is one of the most common endocrinopathies in 5-18% of premenopausal women, which is characterized by hyperandrogenism and ovulatory dysfunction (6). It has been reported that insulin resistance (IR) may be involved in pathophysiological aspect of PCOS, but the exact mechanism of PCOS has not been fully understood yet (7). IR is characterized by a decrease in cellular ability to respond to insulin signaling and known as a basic pathophysiological mechanisms in development of metabolic complications of PCOS (8). 

Non-alcoholic fatty liver disease (NAFLD) is the most common cause of abnormal liver enzymes (9). IR of adipose tissue and reduced whole-body insulin sensitivity are induced by NAFLD. The levels of inflammatory cytokines, like interleukin-6 (IL-6) and tumor necrosis factor-alpha (TNF-α), as well as C-reactive protein (CRP) are elevated in obesity and insulin-resistant states. Furthermore, it has been demonstrated that the plasma levels of these two inflammatory cytokines are increased in subjects with NAFLD and nonalcoholic steatohepatitis (NASH), whereas peripheral blood monocyte productions of TNF-α and IL-6 are increased in subjects with NASH (10). Due to influence of NAFLD and PCOS on fertility, it is very crucial to control these dysfunctional activities of liver for giving the best outcomes in the fertility life of females. CRP is also known as a classic index of low-grade inflammation with a quantity increasing rapidly in the blood circulation and an independent indicator of major cardiac- cardiovascular complications (11). Considering the effects of metabolic complications of PCOS on liver, as well as anti-inflammatory effects of curcumin via modulation of cytokines and IR, this study aimed to investigate the therapeutic effects of curcumin on IL-6 and CRP levels as well as IR index on liver function in PCOS rats. 

## Materials and Methods

This experimental study was conducted at the Department of Cell and Molecular Biology of Kharazmi University (Iran). About 90 adult female Wistar rats weighing 170 ± 20 g were obtained from the animal house of the Kharazmi University. The animals were kept under a 12- hour light/12-hour dark cycle. All research animals were treated in compliance with the guidelines approved by the Kharazmi University in accordance with the National Institutes of Health (NIH) Guidelines for the Care and Use of Laboratory Animals (C: 616/9111). In this experiment, adult female Wistar rats with a 2-3 regular estrous cycle period within a twelve- to fourteen-day period were used. PCOS rats were selected based on displaying a minimum of two continuous estrous cycles. Rats were in the estrous stage of their reproduction cycles. The rats were divided into four groups as follows: control (n=18), sham (n=18), PCOS (n=18) and curcumin-treated PCOS (n=36) groups. For hormonal indication, 2 mg/kg sesame oil- solved estradiol valerate (E2V) was injected subcutaneously to sham, PCOS and curcumin- treated PCOS groups. After injection, vaginal smear test was performed daily. The test was stop when a series of changes in estrus cycle started. The animals reached the stage of persistent vaginal cornification (PVC), which is usually 60 days after injection of E2V. In this study, 2000 mg/kg body weight (BW) curcumin were determined as the concentration of LD50 that is defined as the concentration of lethal dose causing death in 50% of mice (12). Curcumin as a lipophilic polyphenol is insoluble in water, but is readily soluble in organic solvents such as dimethyl sulfoxide (DMSO, Sigma, USA) (13). Therefore, a stock solution of curcumin (Sigma, USA) was prepared at 100 mmol/L in DMSO. The concentrations of 100 and 300 mg/kg BW were then selected as the treatment. After 14 consecutive days of treatment with curcumin, the rats were killed by chloroform inhalation. Then, a part of the right lobe of the liver was removed and fixed in alcoholic Bouin’s solution (Merck, Germany) for histological analysis. Fixed samples were kept in alcohol solutions of 20 to 100% for a period of 45 minutes for dehydration, washed in alcohol/xylene (50:50) and xylene for 3 times, and finally blocked in paraffin. Then, 7 µm sections were provided by microtome and placed on gelatin lams. Paraffin was removed from the samples, while the alcohol solutions with different degrees were used for preparing the samples for hematoxylin and eosin (H&E) staining. 

### Interleukin-6 assay

Serological analysis was performed to measure serum IL-6 levels and hormonal alterations. Serum levels of IL-6 were determined by the enzyme- linked immunosorbent assay (ELISA, rat IL-6 platinum ELISA®, Bender MedSystems, Austria) according to the manufacturer’s instructions. All samples were analyzed in one assay. 

### C-reactive protein measurement

After taking blood samples from hearts of the rats and preparing blood serum using an ELISA kit (Millipore’s MILLIPLEX® MAP Rat/ Mouse CRP Single Plex USA), CRP contents were measured. 

### Collagen special stain (Masson’s trichrome staining)

For liver staining, Masson’s trichrome stain is very common with blue appearance of collagens and red appearance of hepatocytes or other structures of liver. Type 1 collagen is normally seen in portal system and layers of vessels, so this staining protocol is useful in injury conditions like perisinusoidal fibrosis associated with steatohepatitis and periductal fibrosis in primary sclerosing cholangitis (PSC) (14). The trichrome stain is very functional in detection of stages of liver disorder and percentage of liver treatment. Liver tissue slides were placed in staining jar and deparaffinised by submerging into three series of absolute xylene for 4 minutes that was followed by 100, 95, 90, 80 and 70% of ethanol for 4 minutes. The slides were then submerged in warmed Bouin’s solution at 60˚C for 45 minutes, while the slides were rinsed by water to wash out yellow color. To differentiate nuclei, slides were immersed in modified Weigert’s iron haematoxylin (Sigma, USA) for 8 minutes and then, washed in running water for 2 minutes. Cytoplasms of erythrocytes were stained using anionic dyes. Firstly, acid fuschin (Merck, Germany) was applied for 5 minutes, washed out by running water for 2 minutes, and treated with phosphomolybidic acid solution (Merck, Germany) for another 10 minutes. Secondly, the slides were stained by methyl blue (Merck, Germany) solution for 5 minutes in order to stain fibroblast and collagen, washed in running water for 2 minutes, and lastly treated with 1% acetic acid solution for 1 minute. Slides were then dehydrated into a series of alcohol of 70, 80, 95 and 100% for 1 minute in each percentage. Before observation, slides were dipped into absolute xylene for 1 minute and mounted with cover slip using dibutyl phthalate in xylene (DPX, Merck, Germany). Blood samples were evaluated serologically. To accomplish this, the blood sample (about 2 ml) was taken from the animal’s heart and incubated at 37˚C. Serum of blood was prepared by 1500 rpm centrifuge for 10 minutes. It is important to keep insulin (Ultrasensitive ELISA, ALPCO Diagnostics, USA) and glucose (Glucose Oxidase Analyzer, Beckman, USA) at -20˚C when using CHOD- PAP/endpoint method (Merck, Germany). The homeostatic model assessment values for insulin resistance (HOMA-IR) and percent β-cell function (HOMA-β%) were calculated. HOMA-IR was calculated using the formula, as described by Matthews et al. (15), while HOMA-β% was calculated for insulin secretion ability of pancreatic β-cells in different stages of cirrhosis (16). 

HOMA-IR=[fasting insulin (μU/mL)×fasting glucose (mmol/L)/22.5]

HOMA-β%=[20×insulin (μU/mL/glucose mg/ dL)-3.5]

Insulin sensitivity check index (QUICKI) is an empirically-derived mathematical change of fasting blood sugar (FBS) and plasma insulin components. In fact, it is a discrepancy of HOMA equations, through which insulin distributions are slightly skewed (17). 

QUICKI=1/[log (insulin μU/mL)+log glucose mg/ dL)] 

### Statistical analysis

The one-way analysis of variance (ANOVA) test was applied to clarify significant differences among groups. All analyses were performed using InStat version 3 (GraphPad Software, USA). Statistically significant difference was defined as the P<0.05. Furthermore, the corresponding graphs were plotted using EXCEL program. 

## Results

As there was no significant difference between the sham and control groups, we showed only the control data. The results of this study indicated a significant increase in the levels of insulin and blood glucose in PCOS groups as compared to the control group, while in curcumin-treated PCOS groups (100 and 300 mg/kg BW), insulin level significantly reduced (P<0.001 and P<0.05, respectively). In control group, PCOS and curcumin-treated PCOS groups (n=18), serum glucose and insulin levels as well as HOMA-IR were calculated. Obtained results indicated that HOMA-IR in the PCOS group significantly increased as compared to the control group (P<0.001), whereas in the curcumin-treated PCOS groups (100 and 300 mg/kg BW), HOMA-IR decreased significantly as compared to the PCOS group (P<0.001). The HOMA-β% in PCOS group also decreased as compared to control group. QUICKI value in PCOS group had a non-significant decrease as compared to the control group, but in curcumin- treated PCOS groups (300 mg/kg), this value had a significant increase as compared to the PCOS group (P<0.001, Table 1). 

### Interleukin-6 and C-reactive protein assay

**Fig.1:**
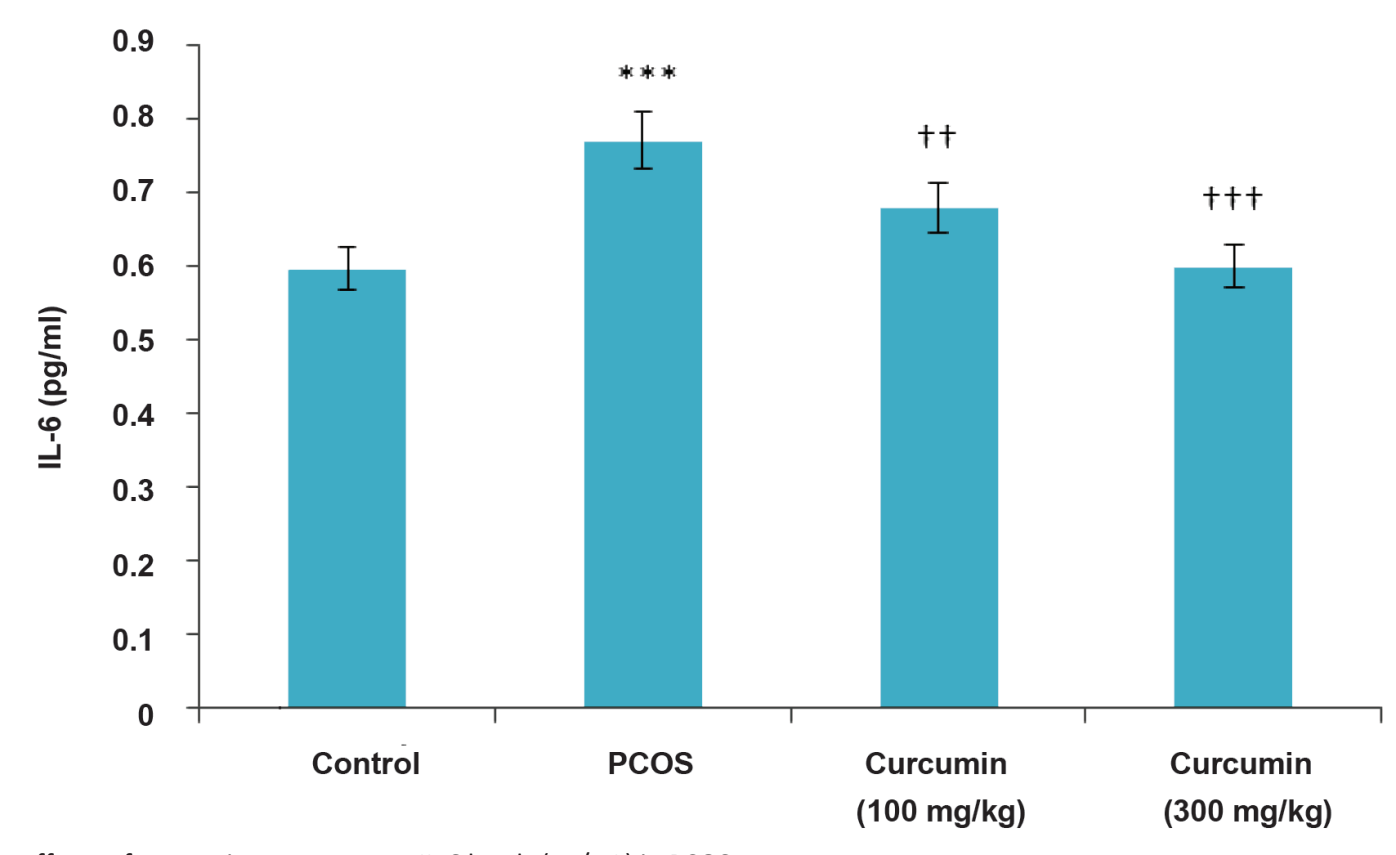
The effects of curcumin treatment on IL-6 levels (pg/mL) in PCOS. PCOS; Polycystic ovary syndrome, IL-6; Interleukin-6, ***; P<0.001 in PCOS vs. control, ††: P<0.01, and †††; P<0.001 in: curcumintreated groups vs. PCOS(n=18).

**Fig.2:**
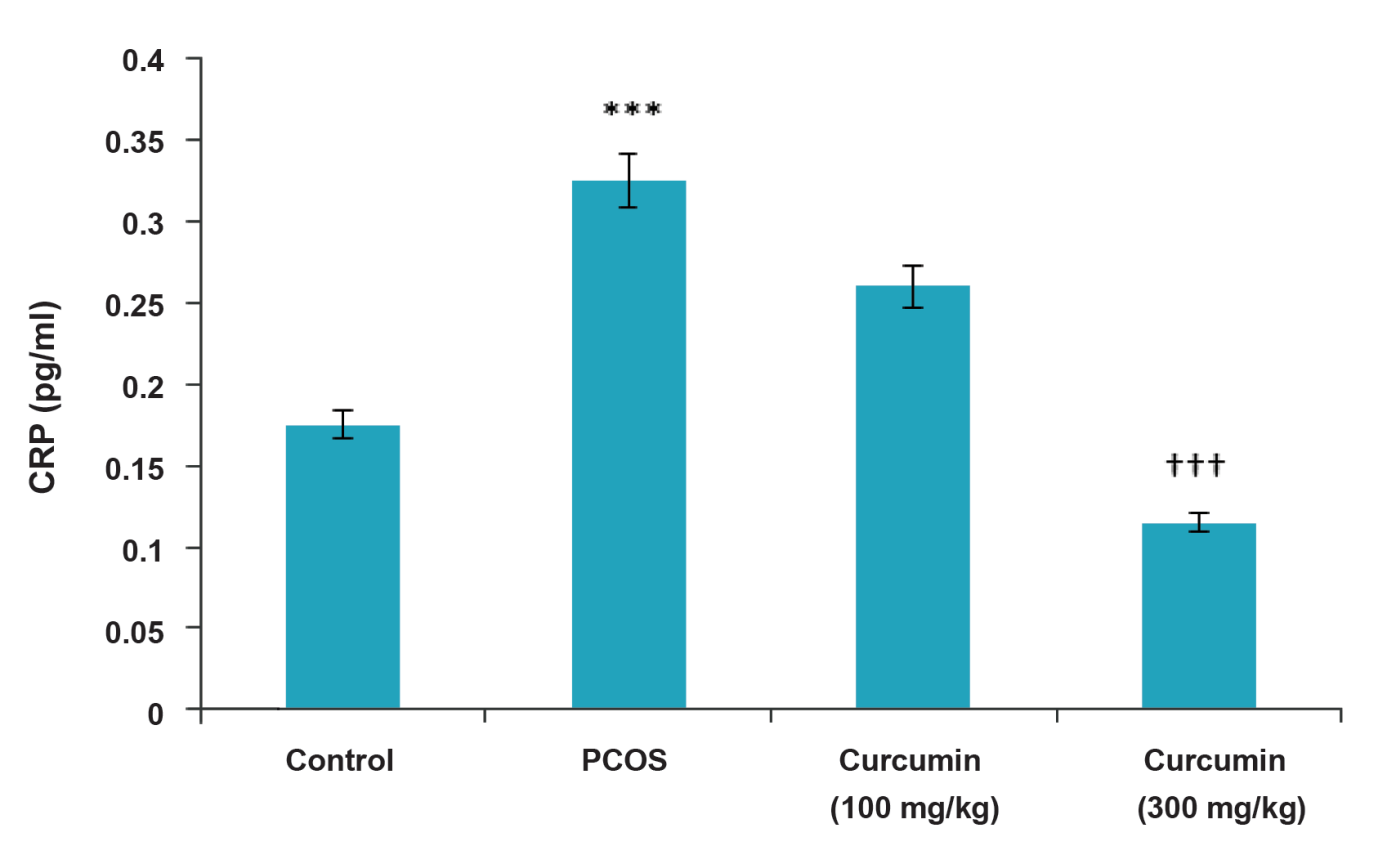
PCOS induction leads to a significant raise in CRP level as compared to the related value of control rats. The effects of curcumintreatment (100, 300 mg/kg BW) on CRP levels (pg/mL) in PCOS. PCOS; Polycystic ovarian syndrome, CRP; C-reactive protein, BW; Body weight, ***; P<0.001 in the PCOS vs. control, and †††; P<0.001 in the curcumin- treated groups vs. PCOS.

In this study, PCOS induction led to a significant rise in IL-6 inflammatory index (P<0.001) as compared to the control rats. The effects of curcumin on IL-6 levels in the PCOS rats were examined for 14 days after the induction of PCOS was completed. As shown in Figure 1, the respective IL-6 levels in the control, PCOS and curcumin-treated PCOS rats (100 and 300 mg/kg BW) were 0.59, 0.77, 0.69 and 0.6 pg/mL, respectively. The IL-6 levels in the curcumin-treated rats (100 and 300 mg/ kg BW) were reduced (P<0.01 and P<0.001, respectively) as compared to the PCOS rats. Our results also showed that administration of curcumin significantly reduced the IL-6 levels in comparison with the related value in the PCOS group (Fig .1). PCOS induction led to a significant raise (P<0.001) in CRP level as compared to the control group, whereas there was a significant reduction in CRP level in the curcumin-treated rats (300 mg/kg BW) as (P<0.001) compared to the PCOS group (Fig .2). 

**Table 1:** Comparison of the glucose and insulin levels as well as HOMA-calculated IR among the control, PCOS and curcumin-treated (100 and 300 mg/kg) groups (mean ± SD)


Group	Glucose (mm/L)	Insulin (U)	HOMA-IR	HOMA-B%	QUICKI

Control	19 ± 0.05	26 ± 0.05	22 ± 0.15	33 ± 0.05	20 ± 0.1
PCOS	25 ± 0.1	33 ± 0.1^*^	36 ± 0.15^***^	30 ± 0.1	12 ± 0.1
Curcumin-treated 100 mg/kg	22 ± 0.1	27 ± 0.1^†^	26 ± 0.1^†††^	29 ± 0.11	16 ± 0.1
Curcumin-treated300 mg/kg	17 ± 0.1	13 ± 0.1^†††^	10 ± 0.15^†††^	19 ± 0.2	35 ± 0.1^†††^


PCOS; Polycystic ovary syndrome, ***; P<0.001, *; P<0.05 in the PCOS vs. control, †††; P<0.001, †; P<0.05 in the curcumin-treated groups vs. PCOS, HOMA-IR; Homeostatic model assessment values for insuline resistance, HOMA-β%; Percent β-cell function, and QUICKI; Quantitative insuline-sensitivity check index.

### H&E staining

**Fig.3:**
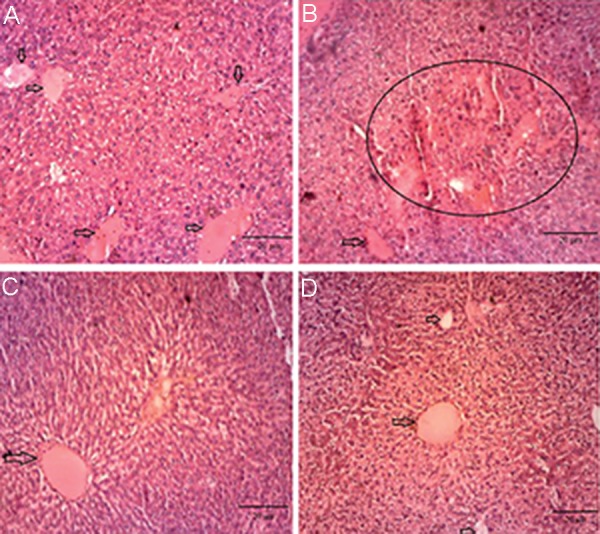
Photomicrograph of healthy liver tissue in control, PCOS and curcumin-treated PCOS groups. A. Control liver, black arrows indicate hepatocytes with a clear border between them, B. PCOS liver, Black circle indicates degeneration hepatocytes with swollen as compared to control groups, C. Liver are treated with various concentrations of the curcumin (100, 300 mg/kg BW) and hepatocytes are in swollen state, and D. Sinusoids are detected and the number of damaged hepatocytes are decreased as compared to PCOS group. PCOS; Polycystic ovarian syndrome, BW; Body weight, H&E; Hematoxylin and eosin staining, (magnification: ×250, scale bar: 20 μm).

After the liver sections were stained by H&E, the control group showed normal results for liver hepatocytes, hepatic lobules, as well as all the three ports (Fig .3A), whereas in the PCOS group, tissue necrosis and hyperemia were observed (Fig .3B). In the curcumin-treated rats (100 mg/kg BW), we found tissue necrosis and core crumpled to a less extent (Fig .3C). In addition, in the curcumin- treated rats (300 mg/kg BW), there were the smooth cords of hepatocytes with the presence of cell boundaries (Fig .3D). Our histological findings indicated that treatment with curcumin (300 mg/ kg BW) exerted the beneficial effects in the PCOS patients with NAFLD (Fig .3). 

### Masson’s trichrome staining

**Fig.4:**
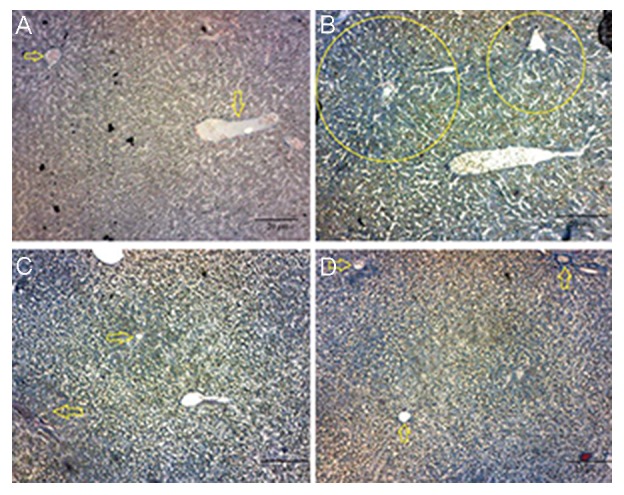
Photomicrograph of the normal wounded liver is stained with modified Masson’s Trichrome staining in polycystic ovarian syndrome (PCOS) and curcumin-treated PCOS groups. A. In control group, there is less collagen expression in Kupffer cells and hepatocytes, B. In PCOS groups, there is more collagen expression in cells fibrosis. Yellow circles indicates degeneration hepatocytes with swollen as compared to control groups, C. In PCOS-treated with curcumin (100 mg/kg), there is less collagen expression in liver cells, and D. In PCOS-treated Curcumin (300 mg/kg), there is no collagen expression in liver cells (magnification: ×40, scale bar: 20 μm). Yellow arrows indicate hepatocytes with a clear border between them.

Our findings showed that in the control group, there was less collagen expression in Kupffer cells and hepatocytes as compared to the PCOS group. However, in PCOS group, there was more collagen expression in cells fibrosis. In the curcumin-treated PCOS rats (100 mg/kg), there was less collagen expression in liver cells, but in the curcumin- treated PCOS rats (300 mg/kg), there was no collagen expression in liver cells (Fig .4). 

## Discussion

In this study, the effects of curcumin on metabolic parameters such as IR, glucose tolerance, IL- 6, CRP and changes of the liver tissue were examined in PCOS-induced rats. IR is regarded as the main factor between metabolic syndrome and PCOS. PCOS patients are known to have a high incidence of IR and glucose intolerance that are associated with higher risk of hypertension and, diabetes mellitus (DM) (18). Wang and Wang (19) have suggested that the role of regulating factors in insulin receptor signaling, like serine/threonine kinase phosphorylating serine residues of IR β-subunit, is very crucial in induction of IR. In order to investigate a possible relationship between IL-6 and hyperandrogenemia with anthropometric or metabolic changes, and gain further insight into the association between PCOS with endocrine and metabolic abnormalities, we measured both IL-6 and CRP levels as parameters of chronic inflammation in PCOS rats. 

It has been shown that increased levels of glucose and insulin are associated with high blood levels of triglycerides and CRP (20). Ibáñez et al. (21) have reported the possible synergistic effects of obesity, IR and DM on the chronic low-level inflammation, which may play a crucial role in the pathogenesis of atherosclerosis, and the possible consideration of CRP as a novel therapeutic target. In a study by ELMekkawi et al. (22), they have showed that three months treatment with metformin reduced body mass index (BMI) and serum IL-6 and IL-18 levels in patients with PCOS. In a study by Elwakkad et al. (23), they have improved anti-inflammatory effects of caffeine with epigallocatechin (EGCG) or green tea extract on the low-grade inflammation state by decreasing the levels of TNF-α, IL-6 and CRP. Nabiuni et al. (24) have also suggested curcumin as an antioxidant/anti-inflammatory agent for improvement of PCOS and initiation of ovulation. Arun and Nalini (25) have showed lower blood glucose and glycated hemoglobin levels in curcumin-treated diabetic rats. Furthermore, they have demonstrated reduced reactive oxygen species (ROS) levels in cells isolated from diabetic patients after curcumin therapy. Seo et al. (26) have also suggested curcumin as an effective agent in improving glucose homeostasis and insulin resistance in db/db mice, leading to activation of glycolysis, inhibition of gluconeogenic and lipid metabolic enzymes in liver, as well as increased activity of lipoprotein lipase (LPL) in skeletal muscle. 

In addition, due to reduction of oxidative stress, curcumin is most likely to be beneficial in preventing diabetic complication. Hala et al. (27) have reported that the mixture of curcumin and ginger used in diabetic rats is effective on both reduction of hyperglycemic and hyperlipidemic and alleviation of oxidative stress. Metformin has been prescribed for PCOS patients since 1994. This drug decreased circulating insulin levels by improvement of peripheral tissues to insuline. This drug also has a beneficial effect on glucose and lipid metabolism that leads to an increase in in the sex hormone binding globulin (SHBG) level and a decrease in androgen level (28). Similarly, our findings indicated that IR is likely to be reduced in PCOS rats treated with different concentration of curcumin (100 and 300 mg/kg). 

In a study by Stephanie et al. (29), they have suggested a molecular mechanism through which curcumin inhibits regulation of IL-6 mRNA expression in LPS-induced vascular smooth muscle cells *in vitro* that results into the inhibition of phosphorylated c-Jun N-terminal kinases (JNK), p38 and extracellular signal-regulated kinase ERK1/2 signaling. Another study has showed that curcumin inhibits the expression induction of COX2, iNOS kinase (JAK-STAT) signaling and cytokines, such as gamma interferon (IFN-gamma) and IL-6 (30). Curcumin also inhibits NF-kappa B (NF-кB) activation induced by TNF, phorbol ester, hydrogen peroxide, or IL-1 in several cell lines. The inhibitory effect of curcumin on NF- кB (nuclear factor kappa-light-chain-enhancer of activated B cells) activation is believed to be due to the inhibition of I-кB kinase (IKK) activity. In multiple myeloma and melanoma cells, curcumin down-regulates NF-кB and prevents nuclear translocation of p65 through the suppression of IKK activity. Therefore, down-regulated NF- кB decreases the expression of inflammatory enzymes, such as COX-2 and iNOS. 

Furthermore, curcumin inhibits NF-кB activation in ovarian cancer cells (31). In current study, immunohistochemical results of liver treated with curcumin showed a significant decrease in the levels of TNF-α, playing an important role in liver. However, there are other inflammatory markers closely related to the PCOS that are needed to be considered, so further studies are required on the association between infertility and inflammatory feature of PCOS. 

## Conclusion

In this study, the total effects of curcumin on metabolic factors in PCOS-induced rats were evaluated for the first time. Our results indicated that curcumin with the ability in adjusting lipid profile and increasing the sensitivity to insulin due to presence of flavonoid may be considered as a natural effective compound to improve some metabolic syndrome in patients with diabetes type 2, and/or PCOS. 
